# A Comparison Between Effects of Amenamevir and Famciclovir on Intensities of Acute Pain and the Incidence of Postherpetic Neuralgia in Adult Patients with Herpes Zoster

**DOI:** 10.14789/jmj.JMJ21-0036-OA

**Published:** 2022-04-15

**Authors:** YUKAKO KAGESHIMA, EIICHI INADA, KEISUKE YAMAGUCHI, MASAKAZU HAYASHIDA

**Affiliations:** 1 Department of Anesthesiology and Pain Medicine, Juntendo University Graduate School of Medicine, Tokyo, Japan; 1 Department of Anesthesiology and Pain Medicine, Juntendo University Graduate School of Medicine, Tokyo, Japan

**Keywords:** amenamevir, famciclovir, herpes zoster, postherpetic neuralgia, zoster associated pain

## Abstract

**Objective:**

Herpes zoster (HZ) is a common disease, whose most common complication is postherpetic neuralgia (PHN). We conducted this study to compare effects of amenamevir (AMNV) and famciclovir (FCV) on intensities of acute HZ pain and the incidence of PHN, which have not been compared yet.

**Methods:**

After approval by the Ethics Committee, we retrospectively investigated adult patients with HZ treated with AMNV or FCV at Juntendo University Hospital between October, 2018 and February, 2020. We compared, between 143 AMNV-treated and 131 FCV-treated patients, pain scores of acute HZ pain evaluated on an 11-point numerical rating scale (NRS) and the incidence of PHN with the Mann-Whitney *U* test and Pearson’s chi-square test, respectively. The univariate logistic regression analysis was used to identify predictors of PHN.

**Results:**

Pain scores during the acute HZ period remained significantly lower in AMNV-treated patients than FCV-treated patients (*p* = 0.049, 0.011, and 0.016 for Day 3-4, Day 7, and Week 2-3, respectively), although the pain score at Day 0 before treatment didn’t differ between them (*p* > 0.05). The incidence of PHN didn’t differ between them (9.8% *vs*. 11.5%, *p* > 0.05). In the total cohort, the pain score at Week 2-3 was significantly associated with the development of PHN (*r*^2^ = 0.180, *p* < 0.00001).

**Conclusions:**

Compared with FCV, AMNV was more effective in reducing acute HZ pain, possibly reflecting its unique mechanism of action. However, AMNV didn’t reduce the incidence of PHN possibly due to the multifactorial etiology of PHN.

## Introduction

Twenty-five percent of the whole population is affected by Herpes Zoster (HZ), of which the most common complication is postherpetic neuralgia (PHN)^1)^. Famciclovir (FCV) is a guanine analog drug used as an antiherpetic agent, whose dose is not affected by renal function, unlike older drugs including valaciclovir (VACV)^2-4)^. Reportedly, FCV prevents acute HZ pain more effectively than VACV^5)^. Amenamevir (AMNV) is a newly-developed helicase-primase inhibitor, which is capable of preventing formation of new skin lesions more effectively without producing worse side effects, compared with VACV^6)^. To date, however, the effects of the newest antiherpetic agent AMNV and the second newest antiherpetic agent FCV on intensities of acute HZ pain and the incidence of PHN have not been compared. Therefore, we conducted the present study to compare the effects of AMNV and FCV on these two endpoints.

## Methods

Prior to this retrospective, observational study, the study protocol was reviewed and approved by the Ethics Committee of Juntendo University Hospital (approval number, 20-015) with a waiver of patients’ written informed consent.

## Patients

We accessed the hospital medical record system and identified all adult male or non-pregnant female patients who visited any outpatient department of Juntendo University Hospital - a 1051-bed university-affiliated hospital in Tokyo, Japan, and who were diagnosed with HZ between October, 2018 and February, 2020. Among them, we investigated patients who were treated with either AMNV or FCV. Excluded were patients who did not complete the initial 7-day session of the antiherpetic agent therapy for any reason, and patients whose pain scores evaluated on an 11-point numerical rating scale (NRS) (0 = no pain, 10 = worst pain) during the acute HZ period were not thoroughly recorded on medical records.

## Data collection

From the medical record, we collected data on baseline clinical characteristics, such as patients’ demography, including age and sex; antiherpetic agents administered, including AMNV and FCV; the time from onset of symptoms/signs of HZ to the initiation of antiherpetic agent therapy; patients’ conditions prior to onset of HZ, including administration of systemic antibiotics for any bacterial infection, daily uses of systemic steroids for underlying diseases, other immunocompromised states, and daily uses of antidepressants; and location of rashes. Immunocompromised patients other than steroid users were defined as patients receiving chemotherapy and/or radiotherapy for cancer. Although antidepressants have not been listed as a predisposing factor for HZ, we included them in clinical characteristics because they might affect intensities of acute HZ pain through their well-known analgesic effects on PHN^1)^. We also collected data on primary clinical outcomes associated with intensities of acute HZ pain, including non-steroidal anti-inflammatory drugs (NSAIDs) required to relieve acute HZ pain; the NRS pain score reported by patients at Day 0 just prior to the initiation of antiherpetic agent therapy, and NRS pain scores at Day 3-4, Day 7, and Week 2-3 after initiating the therapy. Further, we collected data on secondary clinical outcomes associated with development of PHN as defined as pain lasting for 6 months or more^7-9)^, including the incidence of PHN and predisposing factors of PHN.

## Study design

To compare effects of AMNV and FCV on primary clinical outcomes, we first compared NRS pain scores of acute HZ pain and requirements of NSAIDs between patients treated with AMNV and those treated with FCV. We then evaluated the effect of the time from onset of HZ symptoms/signs to the initiation of antiherpetic agent therapy by comparing the primary clinical outcomes between early visitors receiving the therapy within 3 days after onset and late visitors receiving it later than 3 days, considering that the early initiation of the antiherpetic agents within 72 hours has been recommended to achieve better clinical outcomes^5, 6, 10, 11)^. We also conducted subgroup analyses by comparing the primary clinical outcomes between AMNV and FCV separately in early visitors and in late visitors.

At the same time, we compared the secondary clinical outcomes including the incidence of PHN between antiherpetic agents and between early and late visitors. We also attempted to identify predictors of PHN.

## Statistical analysis

Variables are, in principle, shown as the Mean ± SD, Median (Interquartile Range), or Number (%) according to data types. Parametric data such as age were compared between dichotomized patient groups using the unpaired *t* test. Nonparametric data such as NRS pain scores were compared between the groups with the Mann-Whitney *U* test. Changes in pain scores were examined with the Wilcoxon signed-rank test followed by the Bonferroni correction. Categorical data were compared between the groups with the Pearson’s chi-square test. Comparisons of clinical outcomes between patient groups were performed also after the nearest neighbor propensity score matching (PSM) in 1:1 ratio was applied to generate a propensity score-matched (PSM) pair of patients with comparable clinical characteristics, using scores calculated with a multivariate logistic regression model based on differences in clinical characteristics. The univariate logistic regression analysis was used to identify predictors of PHN. The receiver-operating characteristic (ROC) analysis was used to obtain a cutoff point to construct categorical data from continuous data. The statistical analysis was performed with StatFlex ver. 7 (ARTECH, Osaka, Japan). A *p* < 0.05 was considered statistically significant, except when a *p* < 0.0083 was considered significant for multiple comparisons among pain scores at four time points.

## Results

## Comparison between AMNV and FCV in the total cohort

**Figure 1:**
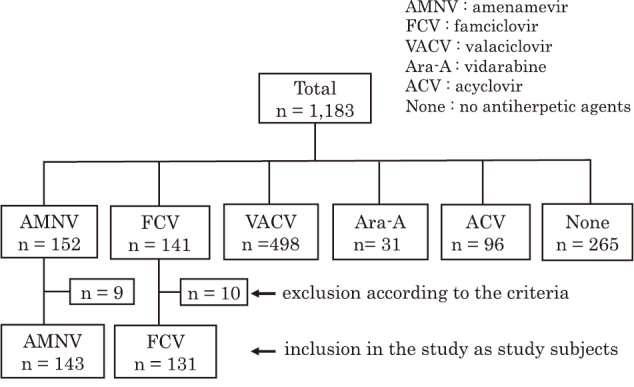
Flow chart for study subject selection

**Figure 2:**
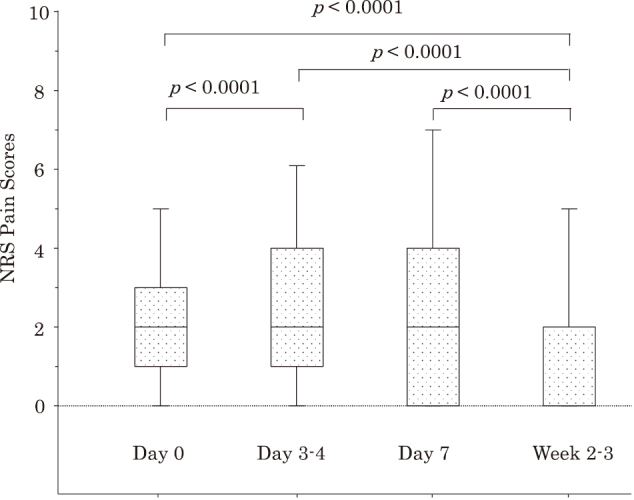
Changes in numerical rating scale (NRS) pain scores during the acute stage of herpes zoster Data are expressed as box and whisker plots. A solid line in the box depicts the median. Ends of the box represent the 75th and 25th percentiles. Whiskers represent the 90th and 10th percentiles.

By searching medical records, we identified a total of 1,183 patients who were diagnosed with HZ. We divided them into six groups based on the antiherpetic agent therapy, including AMNV, FCV, VACV, vidarabine (Ara-A), acyclovir (ACV), and no therapy (Figure 1). Among them, we identified 152 and 141 patients who were treated with AMNV and FCV, respectively. After excluding 9 and 10 patients from AMNV- and FCV-treated patients, respectively, according to the above-mentioned exclusion criteria, we finally investigated a total of 274 patients, including 143 AMNV-treated patients and 131 FCV-treated patients (Figure 1). Baseline clinical characteristics and primary/secondary clinical outcomes in the total cohort are shown in Table 1. In the total cohort, the NRS pain score increased significantly at Day 3-4, but significantly decreased by Week 2-3, compared with Day 0 just before treatment (Figure 2). Seventy patients out of 274 (25.5%) required NSAIDs to relieve acute HZ pain.

**Table 1:** Clinical characteristics and primary/secondary clinical outcomes in the total cohort (n = 274)

Clinical characteristics	
Age (years)	65.3 ± 15.9 (22-98）
Sex; Males and Females	Males, 120 (43.8) / Females, 154 (56.2)
Antiherpetic agents, AMNV and FCV	AMNV, 143 (52.2) / FCV, 131 (47.8)
Early and Late visitors	Early, 150 (54.7) / Late, 124 (45.3)
Antibiotic therapy	27 (9.9)
Steroid user	38 (13.9)
Immunocompromised state	49 (17.9)
Antidepressant user	15 (5.5)
Rash locations	V, 75 (27.4); C, 52 (19.0); TU, 59 (21.5); TL, 43 (15.7); L, 25 (9.1); S, 20 (7.3)
Primary clinical outcomes	
Requirements of NSAIDs	70 (25.5)
NRS pain score at Day 0	2 (1, 3) ［0-10］
NRS pain score at Day 3-4	2 (1, 4) ［0-10］
NRS pain score at Day 7	2 (0, 4) ［0-10］
NRS pain score at Week 2-3	0 (0, 2) ［0-10］
Secondary clinical outcomes	
PHN	29 (10.6)

Data are shown as Mean ± SD (Range), Median (Interquartile Range)［Range］, or Number (%), and compared between patients with unpaired *t* test, Mann-Whitney *U* test, or Pearson's chi-square test. AMNV, amenamevir; FCV, famciclovir; V, trigeminal; C, cervical; TU, upper thoracic (T1-8); TL, lower thoracic (T9-12); L, lumbar; S, sacral; NSAIDs, non-steroidal anti-inflammatory drugs; NRS, numerical rating scale; PHN, postherpetic neuralgia

**Figure 3:**
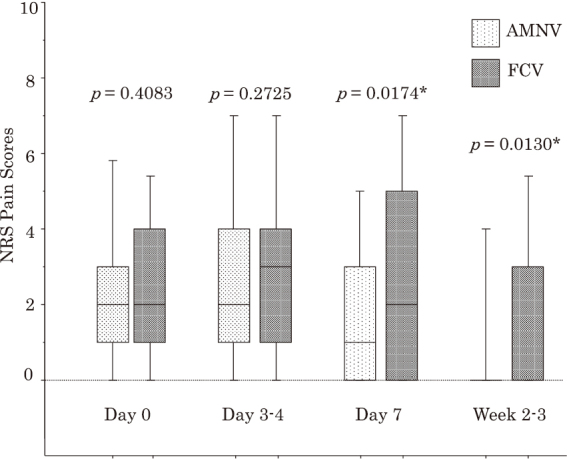
Comparison between numerical rating scale (NRS) pain scores in 111 propensity score-matched pairs of patients treated with amenamevir (AMNV) and patients treated with famciclovir (FCV) Data are expressed as box and whisker plots. A solid line in the box depicts the median. Ends of the box represent the 75th and 25th percentiles. Whiskers represent the 90th and 10th percentiles. * Significant difference between patients

First, we compared clinical characteristics and primary/secondary clinical outcomes between 143 AMNV-treated patients and 131 FCV-treated patients. AMNV-treated patients were significantly younger and included significantly more early visitors (*p* = 0.0094 and *p* = 0.0342, respectively). In AMNV-treated patients, NRS pain scores were significantly lower at Day 3-4, Day 7, and Week 2-3, compared with FCV-treated patients (*p* = 0.0486, *p* = 0.0110, and *p* = 0.0157, respectively), while requirements of NSAIDs did not differ (Table 2). The incidence of PHN did not differ between these patients (Table 2). In 111 PSM pairs with comparable clinical characteristics, NRS pain scores at Day 7 and Week 2-3 were significantly lower in AMNV-treated than FCV-treated patients (*p* = 0.0174 and *p* = 0.0130, respectively), whereas the incidence of PHN did not differ significantly between them (Table 2, Figure 3).

**Table 2:** Comparisons of clinical characteristics and primary/secondary clinical outcomes between patients treated with AMNV and patients treated with FCV before and after propensity score matching (PSM)

Variables	Before PSM			After PSM		
Antiherpetic agents, n	AMNV (n = 143)	FCV (n = 131)	*p* values	AMNV (n = 111)	FCV (n = 111)	*p* values
Clinical characteristics						
Age (years)	62.9 ± 15.4	67.9 ± 16.2	0.0094	66.2 ± 14.3	66.3 ± 16.8	0.9554
Sex; Males and Females	M, 83 (58.0) F, 60 (42.0)	M, 71 (54.2) F, 60 (45.8)	0.5218	M, 47 (42.3) F, 64 (57.7)	M, 51 (45.9) F, 60 (54.1)	0.5888
Early and Late visitors	Early, 87 (60.8) Late, 56 (39.2)	Early, 63 (48.1) Late, 68 (51.9)	0.0342	Early, 60 (54.1) Late, 51 (45.9)	Early, 58 (52.3) Late, 53 (47.7)	0.7879
Antibiotic therapy	15 (10.5)	12 (9.2)	0.7123	9 (8.1)	8 (7.2)	0.8007
Steroid user	24 (16.8)	14 (10.7)	0.1447	13 (11.7)	14 (12.6)	0.8373
Immunocompromised state	29 (20.3)	20 (15.3)	0.2795	18 (16.2)	17 (15.3)	0.8539
Antidepressant user	5 (3.5)	10 (7.6)	0.1326	5 (4.5)	3 (2.7)	0.7215
Rash locations	V, 45 (31.5) C, 21 (14.7) TU, 26 (18.2) TL, 23 (16.1) L, 17 (11.9) S, 11 (7.7)	V, 30 (22.9) C, 31 (23.7) TU, 33 (25.2) TL, 20 (15.3) L, 8 (6.1) S, 9 (6.9)	0.1134	V, 33 (29.7) C, 16 (14.4) TU, 20 (18.0) TL, 19 (17.1) L, 14 (12.6) S, 9 (8.1)	V, 29 (26.1) C, 25 (22.5) TU, 31 (27.9) TL, 14 (12.6) L, 6 (5.4) S, 6 (5.4)	0.1027
Primary clinical outcomes						
Requirements of NSAIDs	37 (25.9)	33 (25.2)	0.8969	26 (23.4)	29 (26.1)	0.6409
NRS pain score at Day 0	2 (1, 3)	2 (1, 4)	0.0905	2 (1, 3)	2 (1, 4)	0.4083
NRS pain score at Day 3-4	2 (1, 3)	3 (1, 4)	0.0486	2 (1, 4)	3 (1, 4)	0.2725
NRS pain score at Day 7	1 (0, 3)	2 (0, 5)	0.0110	1 (0, 3)	2 (0, 5)	0.0174
NRS pain score at Week 2-3	0 (0, 1)	0 (0, 3)	0.0157	0 (0, 0)	0 (0, 3)	0.0130
Secondary clinical outcomes						
PHN	14 (9.8)	15 (11.5)	0.6554	13 (11.7)	13 (11.7)	1.0000

Data are shown as Mean ± SD, Median (Interquartile Range), or Number (%), and compared between patients with unpaired t test, Mann-Whitney U test, or Pearson’s chi-square test. PSM, propensity score matching; AMNV, amenamevir; FCV, famciclovir; M, males; F, females; V, trigeminal; C, cervical; TU, upper thoracic (T1-8); TL, lower thoracic (T9-12); L, lumbar; S, sacral; NSAIDs, non-steroidal anti-inflammatory drugs; NRS, numerical rating scale; PHN, postherpetic neuralgia

## Comparison between early visitors and late visitors

**Figure 4:**
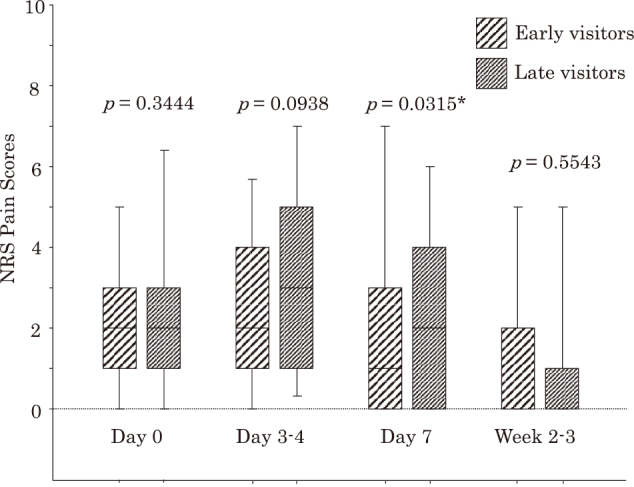
Comparison between numerical rating scale (NRS) pain scores in 108 propensity score-matched pairs of early visitors receiving treatment within 3 days after onset and late visitors receiving treatment later than 3 days Data are expressed as box and whisker plots. A solid line in the box depicts the median. Ends of the box represent the 75th and 25th percentiles. Whiskers represent the 90th and 10th percentiles. * Significant difference between patients

Second, we compared clinical characteristics and primary/secondary clinical outcomes between 150 early visitors and 124 late visitors. AMNV was more frequently used in early visitors than in late visitors (*p* = 0.0342) (Table 3). Requirements of NSAIDs or NRS pain scores did not differ significantly between early and late visitors during the acute HZ period, although pain scores tended to be higher at Day 3-4 and Day 7 in late than early visitors (*p* = 0.0979 and *p* = 0.0671, respectively) (Table 3). In 108 PSM pairs with comparable clinical characteristics, the pain score at Day 3-4 tended to be higher and that at Day 7 was significantly higher in late than early visitors (*p* = 0.0938 and *p* = 0.0315, respectively) (Table 3, Figure 4).

**Table 3:** Comparisons of clinical characteristics and primary/secondary clinical outcomes between early visitors and late visitors before and after propensity score matching (PSM)

Variables	Before PSM			After PSM		
Hospital visit	Early (n = 150)	Late (n = 124)	*p* values	Early (n = 108)	Late (n = 108)	*p* values
Clinical characteristics						
Age (years)	64.8 ± 15.7	66.0 ± 16.3	0.5286	65.4 ± 15.6	65.8 ± 16.3	0.8244
Sex; Males and Females	M, 63 (42.0) F, 87 (58.0)	M, 57 (46.0) F, 67 (54.0)	0.5100	M, 52 (48.1) F, 56 (51.9)	M, 52 (48.1) F, 56 (51.9)	1.0000
Antiherpetic agents, AMNV and FCV	AMNV, 87 (58.0) FCV, 63 (42.0)	AMNV, 67 (54.0) FCV, 57 (46.0)	0.0342	AMNV, 60 (55.6) FCV, 48 (44.4)	AMNV, 53 (49.1) FCV, 55 (50.9)	0.3403
Antibiotic therapy	18 (12.0)	9 (7.3)	0.1899	7 (6.5)	9 (8.3)	0.6033
Steroid user	22 (14.7)	16 (12.9)	0.6742	10 (9.3)	14 (13.0)	0.3865
Immunocompromised state	29 (19.3)	20 (16.1)	0.4909	13 (12.0)	18 (16.7)	0.3319
Antidepressant user	7 (4.7)	8 (6.5)	0.5180	6 (5.6)	8 (7.4)	0.5804
Rash locations	V, 47 (31.3) C, 23 (15.3) TU, 33 (22.0) TL, 21 (14.0) L, 13 (8.7) S, 13 (8.7)	V, 28 (22.6) C, 29 (23.4) TU, 26 (21.0) TL, 22 (17.7) L, 12 (9.7) S, 7 (5.6)	0.3278	V, 27 (25.0) C, 20 (18.5) TU, 25 (23.1) TL, 15 (13.9) L, 12 (11.1) S, 9 (8.3)	V, 27 (25.0) C, 18 (16.7) TU, 26 (24.1) TL, 21 (19.4) L, 10 (9.3) S, 6 (5.6)	0.8619
Primary clinical outcomes						
Requirements of NSAIDs	36 (24.0)	34 (27.4)	0.5183	25 (23.1)	30 (27.8)	0.4349
NRS pain score at Day 0	2 (1, 3)	2 (1, 3)	0.4891	2 (1, 3)	2 (1, 3)	0.3444
NRS pain score at Day 3-4	2 (1, 3)	2.5 (1, 5)	0.0979	2 (1, 4)	3 (1, 5)	0.0938
NRS pain score at Day 7	1 (0, 3)	2 (0, 4)	0.0671	1 (0, 3)	2 (0, 4)	0.0315
NRS pain score at Week 2-3	0 (0, 2)	0 (0, 1)	0.6979	0 (0, 2)	0 (0, 1)	0.5543
Secondary clinical outcomes						
PHN	17 (11.3)	12 (9.7)	0.6574	15 (13.9)	12 (11.1)	0.5371

Data are shown as Mean ± SD, Median (Interquartile Range), or Number (%), and compared between patients with unpaired *t* test, Mann-Whitney *U* test, or Pearson's chi-square test. PSM, propensity score matching; AMNV, amenamevir; FCV, famciclovir; M, males; F, females; V, trigeminal; C, cervical; TU, upper thoracic (T1-8); TL, lower thoracic (T9-12); L, lumbar; S, sacral; NSAIDs, non-steroidal anti-inflammatory drugs; NRS, numerical rating scale; PHN, postherpetic neuralgia

## Comparison between AMNV and FCV in early visitors

Third, based on above-mentioned results, we performed the first subgroup analysis by comparing primary/secondary clinical outcomes between 87 AMNV-treated patients and 63 FCV-treated patients within early visitors. In early visitors, AMNV-treated patients were younger and included more steroid users than FCV-treated patients (*p* = 0.0237 and *p* = 0.0474, respectively) (Table 4), while pain scores during the acute HZ period or the incidence of PHN did not differ between AMNV- and FCV-treated patients, although requirements of NSAIDs were more in AMNV-treated than FCV- treated patients (*p* = 0.0473) (Table 4). In 52 PSM pairs in early visitors with comparable clinical characteristics, these primary or secondary clinical outcomes, including requirements of NSAIDs, did not differ between AMNV- and FCV-treated patients (Table 4).

**Table 4:** Comparisons of clinical characteristics and primary/secondary clinical outcomes between patients treated with AMNV and patients treated with FCV within early visitors before and after propensity score matching (PSM)

Variables	Before PSM			After PSM		
Antiherpetic agents (n)	AMNV (n = 87)	FCV (n = 63)	*p* values	AMNV (n = 52)	FCV (n = 52)	*p* values
Clinical characteristics						
Age (years)	62.3±14.3	68.2±17.1	0.0237	65.6±13.4	66.1±17.8	0.8717
Sex; Males and Females	M, 33 (37.9) F, 54 (62.1)	M, 30 (47.6) F, 33 (52.4)	0.2354	M, 23 (44.2) F, 29 (55.8)	M, 24 (46.2) F, 28 (53.8)	0.8438
Antibiotic therapy	12 (13.8)	6 (9.5)	0.4271	6 (11.5)	5 (9.6)	0.7498
Steroid user	17 (19.5)	5 (7.9)	0.0474	3 (5.8)	5 (9.6)	0.7155
Immunocompromised state	21 (24.1)	8 (12.7)	0.0799	7 (13.5)	7 (13.5)	1.0000
Antidepressant user	2 (2.3)	5 (7.9)	0.1062	2 (3.8)	2 (3.8)	1.0000
Rash locations	V, 29 (33.3) C, 14 (16.1) TU, 14(16.1) TL, 11 (12.6) L, 10 (11.5) S, 9 (10.3)	V, 18 (28.6) C, 9 (14.3) TU, 19 (30.2) TL, 10 (15.9) L, 3 (4.8) S, 4 (6.3)	0.2619	V, 20 (38.5) C, 4 (7.7) TU, 9 (17.3) TL, 7 (13.5) L, 6 (11.5) S, 6 (11.5)	V, 15 (28.8) C, 7 (13.5) TU, 17 (32.7) TL, 9 (17.3) L, 2 (3.8) S, 2 (3.8)	0.1433
Primary clinical outcomes						
Requirements of NSAIDs	26 (29.9)	10 (15.9)	0.0473	13 (25.0)	8 (15.4)	0.2220
NRS pain score at Day 0	2 (1, 3)	2 (1, 4)	0.3267	2 (0.5, 2)	2 (1, 4)	0.1770
NRS pain score at Day 3-4	2 (1, 3)	2 (1, 4)	0.3992	2 (1, 3)	2.5 (1, 4)	0.2361
NRS pain score at Day 7	1 (0, 3)	2 (0, 4)	0.3535	1 (0, 3)	2 (0, 3.5)	0.3293
NRS pain score at Week 2-3	0 (0, 1)	0 (0, 2.5)	0.6190	0 (0, 1)	0 (0, 2)	0.6817
Secondary clinical outcomes						
PHN	11 (12.6)	6 (9.5)	0.5519	8 (15.4)	5 (9.6)	0.3737

Data are shown as Mean ± SD, Median (Interquartile Range), or Number (%), and compared between patients with unpaired *t* test, Mann-Whitney *U* test, or Pearson's chi-square test. PSM, propensity score matching; AMNV, amenamevir; FCV, famciclovir; M, males; F, females; V, trigeminal; C, cervical; TU, upper thoracic (T1-8); TL, lower thoracic (T9-12); L, lumbar; S, sacral; NSAIDs, non-steroidal anti-inflammatory drugs; NRS, numerical rating scale; PHN, postherpetic neuralgia

## Comparison between AMNV and FCV in late visitors

**Figure 5:**
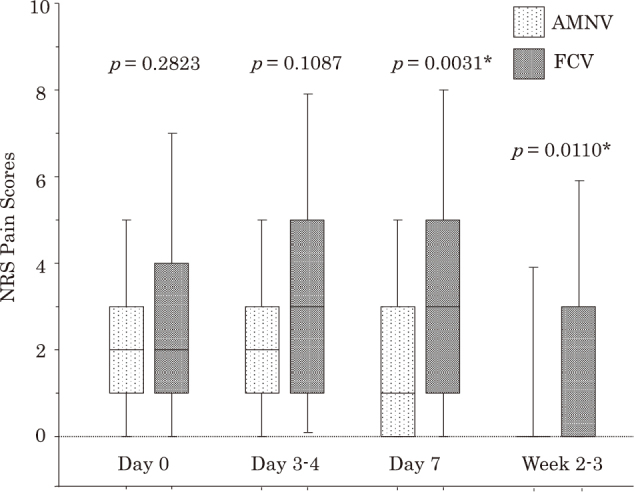
Comparison between numerical rating scale (NRS) pain scores in 46 propensity score-matched pairs of patients treated with amenamevir (AMNV) and patients treated with famciclovir (FCV) in late visitors Data are expressed as box and whisker plots. A solid line in the box depicts the median. Ends of the box represent the 75th and 25th percentiles. Whiskers represent the 90th and 10th percentiles. * Significant difference between patients

Forth, we performed the second subgroup analysis by comparing primary/secondary clinical outcomes between 56 AMNV-treated patients and 68 FCV-treated patients within late visitors. In late visitors, requirements of NSAIDs tended to be less, the pain score at Day 3-4 tended to be lower, and pain scores at Day 7 and Week 2-3 were significantly lower in AMNV-treated than FCV-treated patients (*p* = 0.0782, *p* = 0.0904, *p* = 0.0015 and *p* = 0.0111, respectively), although the incidence of PHN did not differ between them (Table 5). In 46 PSM pairs in late visitors with more comparable clinical characteristics, pain scores at Day 7 and Week 2-3 were significantly lower (*p* = 0.0031 and *p* = 0.0110, respectively), and the incidence of PHN tended to be lower (3/46 *vs.* 9/46, *p* = 0.0633), in AMNV-treated than FCV-treated patients, although requirements of NSAIDs for acute pain control did not differ between them (Table 5, Figure 5).

**Table 5:** Comparisons of clinical characteristics and primary/secondary clinical outcomes between patients treated with AMNV and patients treated with FCV within late visitors before and after propensity score matching (PSM)

Variables	Before PSM			After PSM		
Antiherpetic agents, n	AMNV (n = 56)	FCV (n = 68)	*p* values	AMNV (n = 46)	FCV (n = 46)	*p* values
Clinical characteristics						
Age (years)	63.9±17.1	67.7±15.5	0.1960	67.6±15.7	67.3±13.7	0.9211
Sex; Males and Females	M, 27 (48.2) F, 29 (51.8)	M, 30 (44.1) F, 38 (55.9)	0.6487	M, 22 (47.8) F, 24 (52.2)	M, 18 (39.1) F, 28 (60.9)	0.4002
Antibiotic therapy	3 (5.4)	6 (8.8)	0.4591	2 (4.3)	2 (4.3)	1.0000
Steroid user	7 (12.5)	9 (13.2)	0.9033	6 (13.0)	7 (15.2)	0.7647
Immunocompromised state	8 (14.3)	12 (17.6)	0.6125	8 (17.4)	7 (15.2)	0.7778
Antidepressant user	3 (5.4)	5 (7.4)	0.6526	3 (6.5)	1 (2.2)	0.6166
Rash locations	V, 16 (28.6) C, 7 (12.5) TU, 12 (21.4) TL, 12 (21.4) L, 7 (12.5) S, 2 (3.6)	V, 12 (17.6) C, 22 (32.4) TU, 14 (20.6) TL, 10 (14.7) L, 5 (7.4) S, 5 (7.4)	0.1010	V, 13 (28.3) C, 7 (15.2) TU, 9 (19.6) TL, 8 (17.4) L, 7 (15.2) S, 2 (4.3)	V, 9 (19.6) C, 11 (23.9) TU, 13 (28.3) TL, 9 (19.6) L, 1 (2.2) S, 3 (6.5)	0.2132
Primary clinical outcomes						
Requirements of NSAIDs	11 (19.6)	23 (33.8)	0.0782	9 (19.6)	16 (34.8)	0.1009
NRS pain score at Day 0	2 (1, 3)	2 (1, 4)	0.1974	2 (1, 3)	2 (1, 4)	0.2823
NRS pain score at Day 3-4	2 (1, 3.5)	3 (1, 5)	0.0904	2 (1, 3)	3 (1, 5)	0.1087
NRS pain score at Day 7	1 (0, 3)	3 (1, 5)	0.0015	1 (0, 3)	3 (1, 5)	0.0031
NRS pain score at Week 2-3	0 (0, 0)	0 (0, 3)	0.0111	0 (0, 0)	0 (0, 3)	0.0110
Secondary clinical outcomes						
PHN	3 (5.4)	9 (13.2)	0.1398	3 (6.5)	9 (19.6)	0.0633

Data are shown as Mean ± SD, Median (Interquartile Range), or Number (%), and compared between patients with unpaired *t* test, Mann-Whitney *U* test, or Pearson's chi-square test. PSM, propensity score matching; AMNV, amenamevir; FCV, famciclovir; M, males; F, females; V, trigeminal; C, cervical; TU, upper thoracic (T1-8); TL, lower thoracic (T9-12); L, lumbar; S, sacral; NSAIDs, non-steroidal anti-inflammatory drugs; NRS, numerical rating scale; PHN, postherpetic neuralgia

## Predisposing factors of PHN

**Figure 6:**
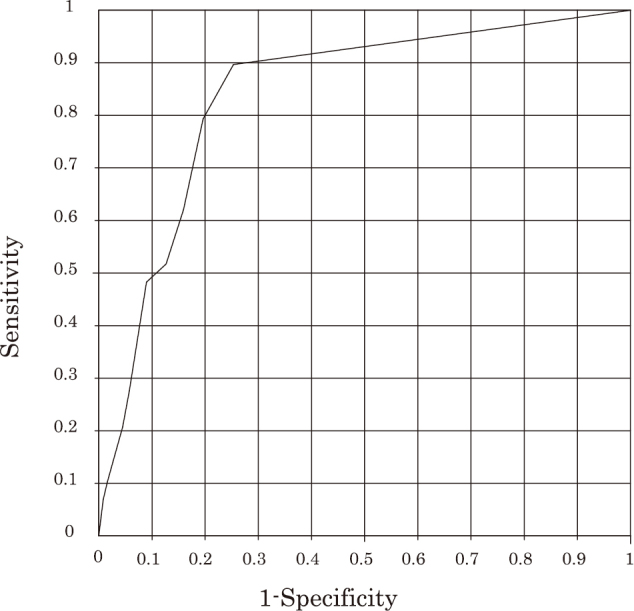
Results of the receiver operating characteristic (ROC) analysis assessing the association between the numerical rating scale (NRS) pain score at Week 2-3 and the development of postherpetic neuralgia (PHN) The area under curve (AUC) and the cutoff point were 0.840 (*p*<0.00001) and 0.9 (sensitivity 80.0%, specificity 80.0%) for the NRS pain score at Week 2-3 predicting an increased risk for PHN.

Fifth, we attempted to identify predisposing factors of PHN as secondary clinical outcomes. In the total cohort, 29 patients out of 274 (10.6%) developed PHN. As a result of the univariate logistic regression analysis, none of clinical characteristics examined, including antiherpetic agents used (i.e. AMNV *vs.* FCV) and the time to the initiations of antiherpetic agent therapy (i.e. early visitors *vs.* late visitors), were not associated with the development of PHN, although older age and steroid users tended to be associated with a higher incidence of PHN (*p* = 0.0714 and *p* = 0.0973, respectively) (Table 6). On the other hand, the development of PHN was significantly associated with some primary clinical outcomes; although the development of PHN was not associated with the pain score at Day 0 or Day 3-4, it was significantly associated with the pain score at Day 7 (*r*^2^ = 0.088, *p* = 0.00006), and even more significantly associated with the pain score at Week 2-3 (*r*^2^ = 0.180, *p* < 0.00001) (Table 6). The ROC analysis revealed that a cutoff point of the NRS pain score at Week 2-3 for predicting an increased risk for PHN was 0.9 (Figure 6). Based on these results, we dichotomized patients into 88 patients with any pain (rated as ≥ 1 on the NRS) at Week 2-3 and 186 patients with no pain (rated as 0 on the NRS) at Week 2-3, and performed the univariate logistic regression analysis, which revealed the odds ratio (OR), 25.6 (95% confidential interval [CI], 7.5-87.5); area under the curve (AUC), 0.822; *r*^2^, 0.257; and a *p* value < 0.00001. The actual incidences of PHN in our patients was 18.3 times higher in patients with any pain at Week 2-3, compared with those with no pain at Week 2-3 (29.5 % [26/88] *vs.* 1.6 % [3/186], *p* < 0.00001).

**Table 6:** Results of comparisons of clinical backgrounds and primary clinical outcomes between patients developing PHN and not developing PHN (＊), and univariate logistic analyses performed in an attempt to identify predictors of PHN (#)

PHN or non-PHN	PHN (n=29)	non-PHN (n=245)	*p* values ＊	*p* values #
Clinical characteristics				
Age (years)	70.4±12.7	64.7±16.2	0.0688	0.0714
Sex; Males and Females	M, 9 (31.0) F, 20 (69.0)	M, 111 (45.3) F, 134 (54.7)	0.1430	0.1476
Antiherpetic agents, AMNV and FCV	AMNV, 14 (48.3) FCV, 15 (51.7)	AMNV, 129 (52.7) FCV, 116 (47.3)	0.6554	0.6557
Early and Late visitors	Early, 17 (58.6) Late, 12 (41.4)	Early, 133 (54.3) Late, 112 (45.7)	0.6574	0.6577
Antibiotic therapy	2 (6.9)	25 (10.2)	0.5720	0.5747
Steroid user	7 (24.1)	31 (12.7)	0.0906	0.0973
Immunocompromised state	6 (20.7)	43 (17.6)	0.6766	0.6771
Antidepressant user	1 (3.4)	14 (5.7)	0.6120	0.6159
Rash locations	V, 8 (27.6) C, 4 (13.8) TU, 6 (20.7) TL, 7 (24.1) L, 2 (6.9) S, 2 (6.9)	V, 67 (27.3) C, 48 (19.6) TU, 53 (21.6) TL, 36 (14.7) L, 23 (9.4) S, 18 (7.3)	0.8312	0.5406
Primary clinical outcomes				
Requirements of NSAIDs	7 (24.1)	63 (25.7)	0.8540	0.8540
NRS pain score at Day 0	2 (1, 3)	2 (1, 3)	0.7130	0.2740
NRS pain score at Day 3-4	2 (1, 6)	2 (1, 4)	0.3460	0.1010
NRS pain score at Day 7	3 (2, 6)	1 (0, 3)	0.00003	0.00006
NRS pain score at Week 2-3	4 (2, 6)	0 (0, 1)	<0.00001	<0.00001

Data are shown as Mean ± SD, Median (Interquartile Range), or Number (%), and compared between patients with unpaired *t* test, Mann-Whitney *U* test, or Pearson's chi-square test (＊). Univariate logistic regression analysis was performed to identify predictors of PHN (#). PHN, postherpetic neuralgia; AMNV, amenamevir; FCV, famciclovir; M, males; F, females; V, trigeminal; C, cervical; TU, upper thoracic (T1-8); TL, lower thoracic (T9-12); L, lumbar; S, sacral; NSAIDs, non-steroidal anti-inflammatory drugs; NRS, numerical rating scale

## Discussion

It has been reported that the elderly over 60 years of age, female sex, and presences of a prodrome, severe rashes, severe acute pain, and/or immunocompromised states are independent predictors of PHN^1, 12-15)^. Because severe acute pain and/or rashes predispose to PHN, earlier and better controls of acute pain and/or rashes achieved with potent antiherpetic agents may help to reduce the incidence of PHN^16)^. Based on this concept, we conducted this study to evaluate whether AMNV is superior to FCV in terms of acute pain control as primary clinical outcomes and prevention of PHN as secondary clinical outcomes.

In the present study, we found that compared to FCV, AMNV was more effective in reducing pain at Day 7 and Week 2-3 in the total cohort both before and after PSM. By performing subgroup analyses, we found that the pain-relieving effects of AMNV and FCV were not different in early visitors receiving antiherpetic agent therapy within 3 days both before and after PSM, whereas AMNV was more effective in reducing acute HZ pain at Day 7 and Week 2-3 in late visitors receiving the therapy later than 3 days both before and after PSM.

Generally, the early initiation of antiviral therapy within 72 hours, and hopefully, within 48 hours, has been recommended for achieving better clinical outcomes, including rapider pain relief ^5, 6, 10, 11, 16)^. Indeed, we found that compared with late visitors, early visitors tended to achieve better pain relief at Day 7 before PSM, and that they achieved significantly better pain relief at Day 7 after PSM. Because the early initiation of antiherpetic agent therapy thus could more effectively relieve acute HZ pain, FCV might exert pain-relieving effects as effective as AMNV in early visitors receiving antiherpetic agent therapy within 3 days after onset of HZ.

On the other hand, AMNV was more effective than FCV in reducing acute HZ pain at Day 7 and Week 2-3 in late visitors receiving the therapy later than 3 days, both before and after PSM. FCV is converted by the liver enzyme to active penciclovir that inhibits viral DNA polymerase in cells infected by the virus^2, 17, 18)^, whereas AMNV directly suppresses viral growth by inhibiting the activity of the helicase-primase complex required for cleavage of double strand DNA and synthesis of RNA primers, which is the initial stage of viral replication^19, 20)^. Considering such pharmacological mechanisms of action quite different between AMNV and FCV, it seems plausible that AMNV has a faster onset of action and a more potent anti-viral effect^6, 21)^, which might explain why AMNV exerted pain-relieving effects superior to FCV in late visitors in our study.

Another major finding of this study was that pain intensities, not in the early phase of acute HZ at Day 0 or Day 3-4, but in the late phase at Day 7 and Week 2-3, especially at Week 2-3, were significantly associated with the development of PHN. Patients with any persisting pain at Week 2-3 was associated with a marked increase in a risk for PHN development, as indicated by a high odds ratio of 25.6. Many previous studies showed significant associations between initial pain intensities upon enrollment in studies and the development of PHN^22-24)^. However, we could not find such associations between initial pain intensities and PHN. Such discrepancies might result from different study designs, such as prospective randomized controlled studies *vs.* a retrospective observational study, and/or the enrollment of early visitors alone *vs.* inclusions of both early and late visitors^22-24)^.

However, our study clearly demonstrated that pain intensities in the late phase, rather than early phase, of acute HZ could be a much more reliable predictor of PHN. Because pain spontaneously resolves with healing of skin rashes within 2-3 weeks in many patients not developing PHN while pain persist beyond such periods in most patients developing PHN lasting for months or more^1, 13, 14)^, it seemed quite reasonable that pain intensities in the late phase of acute HZ, rather than those in the early phase, could be more significantly associated with the development of PHN.

The present study showed that pain scores at Day 7 and at Week 2-3 were significantly associated with the development of PHN, and that AMNV reduced pain at Day 7 and Week 2-3 more effectively than FCV before and after PSM in the total cohort. Therefore, it could be expected that the use of AMNV would lead to a reduced incidence of PHN. In the present study, however, AMNV did not more effectively reduce the incidence of PHN, compared with FCV. Such discrepancies might be explained by the fact that intensities of acute pain account only for a part of the multifactorial etiology of PHN^1, 12-15)^, as was suggested also by the relatively low coefficient of determination (*r*^2^) values provided by univariate logistic regression analyses (0.088 and 0.180 for NRS pain scores at Day 7 and Week 2-3, respectively). As mentioned above, however, AMNV could reduce pain at Day 7 and Week 2-3 more effectively than FCV not in early visitors but only in late visitors both before and after PSM. Further, in PSM pairs of late visitors, AMNV tended to be associated with a lower incidence of PHN, compared with FCV (*p* = 0.0633). Therefore, the possibility could not be completely excluded that compared with FCV, AMNV would reduce pain more effectively in late visitors, thereby reducing the risk of PHN in patients receiving the therapy later than 3 days of HZ onset.

Clearly, our study had limitations resulting from the retrospective, uncontrolled fashion, such as some inaccuracy and/or incompleteness of data, and further studies are required to confirm the pain-relieving effect of AMNV, and to examine whether its use can help to reduce the incidence of PHN.

## Conclusion

AMNV could reduce acute HZ pain more effectively than FCV in late visitors receiving the therapy later than 3 days after onset, but not in early visitors receiving the therapy within 3 days. Intensities of acute HZ pain in the late phase of acute HZ was more predictive of PHN than those in early phase. Further, compared with FCV, AMNV might more effectively reduce the incidence of PHN in late visitors, although this possibility should be confirmed in a further prospective study.

## Funding

No funding was received.

## Author contributions

All authors meet every criteria recommended at the International Committee of Medical Journal Editors (ICMJE). YK collected data, analyzed them using a simple statistical analysis software, and wrote the first draft of the manuscript. EI contributed to conceptualization of this study, reviewed the manuscripts, and helped revise the first draft. KY reviewed the manuscript and advised the authors about additional data collection in improving the manuscript. MH was a major contributor in writing and editing the manuscript and analyzing all of the data. All authors read and approved the final manuscript.

## Conflicts of interest statement

The authors declare that there are no conflicts of interest.
